# (*Z*)-3-(3,4-Dimeth­oxy­benzyl­idene)-2,3-dihydro-1,5-benzothia­zepin-4(5*H*)-one

**DOI:** 10.1107/S1600536813007423

**Published:** 2013-03-23

**Authors:** M. Bakthadoss, R. Selvakumar, N. Manikandan, S. Murugavel

**Affiliations:** a Department of Organic Chemistry, University of Madras, Maraimalai Campus, Chennai 600 025, India; b Department of Physics, Bharathidasan Engineering College, Nattrampalli, Vellore 635 854, India; c Department of Physics, Thanthai Periyar Government Institute of Technology, Vellore 632 002, India

## Abstract

In the title compound, C_18_H_17_NO_3_S, the seven-membered thia­zepine ring adopts a slightly distorted sofa conformation. The dihedral angle between the mean plane of the benzothia­zepine ring system and the benzene ring is 5.9 (1)°. The mol­ecular conformation is stabilized by an intra­molecular C—H⋯S hydrogen bond, which generates an *S*(7) ring motif. In the crystal, N—H⋯O and C—H⋯O hydrogen bonds link inversion-related mol­ecules into dimers, incorporating *R*_1_^2^(6) and *R*_2_^2^(8) ring motifs; the acceptor O atom is bifurcated. These dimers are further linked by C—H⋯O hydrogen bonds, forming supra­molecular tapes running along the *a* axis. These are connected into the three-dimensional architecture by C—H⋯π inter­actions.

## Related literature

For the pharmaceutical properties of thia­zepine derivatives, see: Tomascovic *et al.* (2000 ▶); Rajsner *et al.* (1971 ▶); Metys *et al.* (1965 ▶). For related structures, see: Lakshmanan *et al.* (2012 ▶); Selvakumar *et al.* (2012 ▶); Murugavel *et al.* (2013 ▶). For ring-puckering parameters, see: Cremer & Pople (1975 ▶). For hydrogen-bond motifs, see: Bernstein *et al.* (1995 ▶).
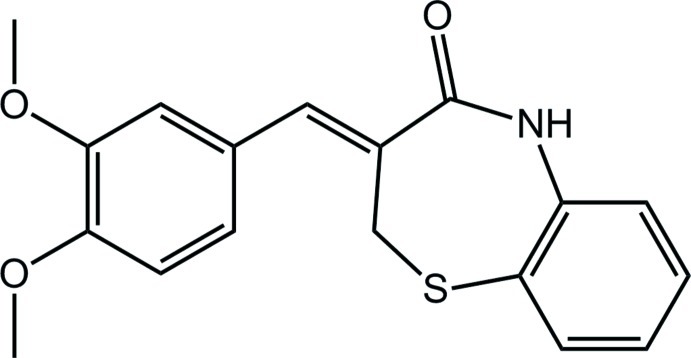


## Experimental

### 

#### Crystal data

C_18_H_17_NO_3_S
*M* *_r_* = 327.39
Triclinic, 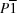

*a* = 7.0249 (4) Å
*b* = 10.7949 (7) Å
*c* = 10.8826 (7) Å
α = 91.783 (3)°
β = 97.562 (2)°
γ = 108.512 (2)°
*V* = 773.42 (8) Å^3^
*Z* = 2
Mo *K*α radiation
μ = 0.22 mm^−1^
*T* = 293 K
0.23 × 0.21 × 0.15 mm

#### Data collection

Bruker APEXII CCD diffractometer
Absorption correction: multi-scan (*SADABS*; Sheldrick, 1996 ▶) *T*_min_ = 0.950, *T*_max_ = 0.967
14052 measured reflections
3082 independent reflections
2545 reflections with *I* > 2σ(*I*)
*R*_int_ = 0.032

#### Refinement

*R*[*F* ^2^ > 2σ(*F* ^2^)] = 0.039
*wR*(*F* ^2^) = 0.121
*S* = 1.06
3082 reflections
210 parameters
H-atom parameters constrained
Δρ_max_ = 0.20 e Å^−3^
Δρ_min_ = −0.27 e Å^−3^

### 

Data collection: *APEX2* (Bruker, 2004 ▶); cell refinement: *APEX2* and *SAINT* (Bruker, 2004 ▶); data reduction: *SAINT* and *XPREP* (Bruker, 2004 ▶); program(s) used to solve structure: *SHELXS97* (Sheldrick, 2008 ▶); program(s) used to refine structure: *SHELXL97* (Sheldrick, 2008 ▶); molecular graphics: *ORTEP-3 for Windows* (Farrugia, 2012 ▶); software used to prepare material for publication: *SHELXL97* and *PLATON* (Spek, 2009 ▶).

## Supplementary Material

Crystal structure: contains datablock(s) global, I. DOI: 10.1107/S1600536813007423/tk5207sup1.cif

Structure factors: contains datablock(s) I. DOI: 10.1107/S1600536813007423/tk5207Isup2.hkl

Supplementary material file. DOI: 10.1107/S1600536813007423/tk5207Isup3.cml

Additional supplementary materials:  crystallographic information; 3D view; checkCIF report

## Figures and Tables

**Table 1:** Hydrogen-bond geometry (Å, °) *Cg*1 is the centroid of the C2–C7 ring.

*D*—H⋯*A*	*D*—H	H⋯*A*	*D*⋯*A*	*D*—H⋯*A*
C12—H12⋯S1	0.93	2.76	3.605 (2)	151
N1—H1⋯O1^i^	0.86	2.12	2.967 (2)	170
C6—H6⋯O1^i^	0.93	2.52	3.318 (2)	144
C5—H5⋯O3^ii^	0.93	2.46	3.377 (3)	167
C17—H17*C*⋯*Cg*1^iii^	0.96	2.84	3.724 (2)	154
C18—H18*A*⋯*Cg*1^iv^	0.96	2.90	3.841 (3)	166

Symmetry codes: (i) 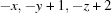; (ii) 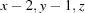; (iii) 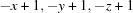; (iv) 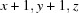.

**Fig. 1.**
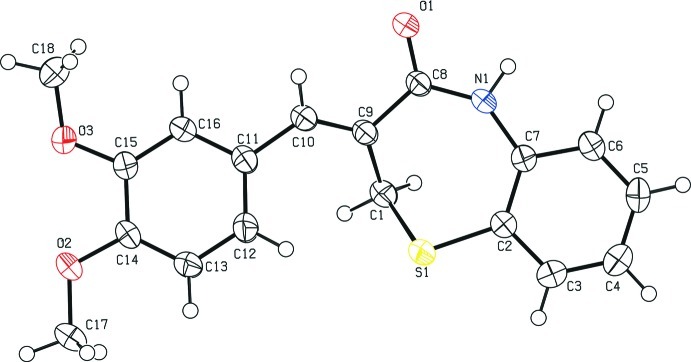
Molecular structure of the title compound showing displacement ellipsoids at the 50% probability level. H atoms are presented as a small spheres of arbitrary radii.

**Fig. 2.**
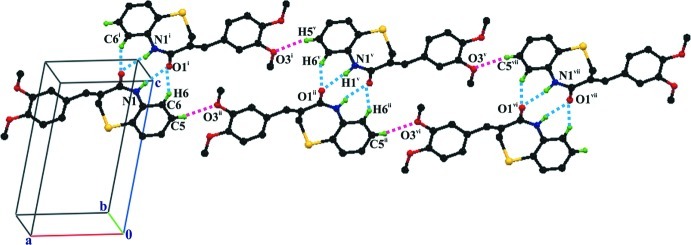
Supramolecular tape formation in the crystal packing of the title compound whereby bifurcated hydrogen bonds link inverted molecules into dimers sustained by N—H···O and C—H···O (cyan dashed lines) contacts are linked via C—H···O contacts (magenta dashed lines) along the a axis [Symmetry codes: (i) -x, 1-y, -z; (v) -2+x, -1+y, z; (v) -2-x, -y, 2-z; (vi) -4+x, -2+y, z; (vii) -4-x, -1-y, 2-z].

**Fig. 3.**
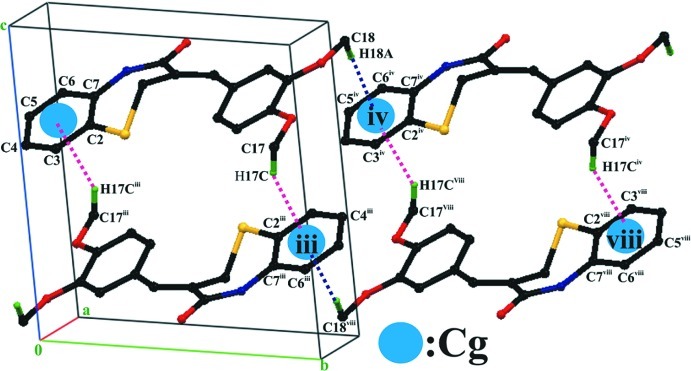
Supramolecular tape formation along the b axis by C—H···π interactions in the crystal structure of the title compound. Cg is the centroid of the (C2–C7) benzene ring. [Symmetry codes: (iii) 1-x, 1-y, 1-z; (iv) 1+x, 1+y, z; (viii) 2-x, 2-y, 1-z].

## supplementary crystallographic information

### Comment

The title compound is used as an intermediate for the synthesis of dosulepin, which is an antidepressant of the tricyclic family. Dosulepin prevents reabsorbing of serotonin and noradrenaline in the brain, helps to prolong the mood lightening effect of any released noradrenaline and serotonin, thus relieving depression. The dibenzo[c,e]thiazepin derivatives exhibit chiroptical properties (Tomascovic *et al.*, 2000). Dibenzo[b,e]thiazepin-5,5-dioxide derivatives possess anti-histamine and anti-allergenic activities (Rajsner *et al.*, 1971). Benzene thiazepin derivatives are identified as a new type of effective anti-histamine compounds (Metys *et al.*, 1965). In view of this biological importance, the crystal structure of the title compound has been carried out and the results are presented here.

Fig. 1. shows a displacement ellipsoid plot of (I), with the atom numbering scheme. The seven membered thiazepine ring (N1/S1/C1/C2/C7/C8/C9) adopts slightly distorted sofa conformation as indicated by puckering parameters (Cremer & Pople, 1975): QT = 0.7878 (17) Å, φ_2_ = 57.8 (2)° and φ_3_ = 16.3 (2)°. The atom O1 deviates by 0.463 (1) Å from the least-squares plane of the thiazepin ring. The dihedral angle between the benzothiazepin ring system and the benzene ring is 5.9 (1)°. The sum of angles at N1 atom of the thiazepin ring (359.9°) is in accordance with *sp*^2^ hybridization. The geometric parameters of the title molecule agree well with those reported for similar structures (Selvakumar *et al.*, 2012, Lakshmanan *et al.*, 2012).

The molecular conformation is stabilized by an intramolecular C12—H12···S1 hydrogen bond, which generates an *S*(7) ring motif (Bernstein *et al.*, 1995). In the crystal, intermolecular bifurcated acceptor N1—H1···O1^i^ and C6—H6···O1^i^ (Table 1) hydrogen bonds link inverted-related molecules into dimers, incorporating *R_1_^2^*(6) and *R_2_^2^*(8) ring motifs. These dimers are further linked by C5—H5···O3^ii^ (Table 1) hydrogen bonds forming supramolecular tapes running along the *a* axis (Fig. 2). The crystal packing is further stabilized by C17—H17C···*Cg* interactions, in which atom C17 acts as a hydrogen bond donor *via* H17C, to a symmetry related C2–C7 benzene ring (Table 1), thereby generating cyclic centrosymmetric dimers. This dimers are further linked by C18—H18A···*Cg*^iv^ (Table 1) interactions into supramolecular tapes running along the *b* axis (Fig. 3 and Table 1; *Cg* is the centroid of the C2–C7 benzene ring).

### Experimental

A mixture of (*Z*)-methyl 2-(bromomethyl)-3-(3,4-dimethoxyphenyl)acrylate 2 mmol) and *o*-aminothiophenol (2 mmol) in the presence of potassium *tert*-butoxide (4.8 mmol) in dry THF (10 ml) was stirred at room temperature for 1 h. After the completion of the reaction as indicated by TLC, the reaction mixture was concentrated and the resulting crude mass was diluted with water (20 ml) and extracted with ethyl acetate (3 *x* 20 ml). The organic layer was washed with brine (2 *x* 20 ml) and dried over anhydrous sodium sulfate. The organic layer was concentrated, which successfully provide the crude final product ((*Z*)-3-(3,4-dimethoxybenzylidene)-2,3-dihydrobenzo[*b*][1,4]). The final product was purified by column chromatography on silica gel to afford the title compound in good yield (46%). Single crystals suitable for X-ray diffraction were obtained by slow evaporation of its ethylacetate solution at room temperature.

### Refinement

All the H atoms were positioned geometrically with C—H = 0.93–0.97 Å and N—H = 0.86 Å and constrained to ride on their parent atom, and with *U*_iso_(H)=1.5*U*_eq_ for methyl H atoms and 1.2*U*_eq_(C) for other H atoms. Owing to poor agreement, one reflection, *i.e*. (0 0 1), was omitted from the final cycles of refinement.

### Crystal data

**Table:**

C_18_H_17_NO_3_S	*Z* = 2
*M**_r_* = 327.39	*F*(000) = 344
Triclinic, *P*1	*D*_x_ = 1.406 Mg m^−^^3^
Hall symbol: -P 1	Mo *K*α radiation, λ = 0.71073 Å
*a* = 7.0249 (4) Å	Cell parameters from 3172 reflections
*b* = 10.7949 (7) Å	θ = 2.0–26.4°
*c* = 10.8826 (7) Å	µ = 0.22 mm^−^^1^
α = 91.783 (3)°	*T* = 293 K
β = 97.562 (2)°	Block, colourless
γ = 108.512 (2)°	0.23 × 0.21 × 0.15 mm
*V* = 773.42 (8) Å^3^	

### Data collection

**Table:**

Bruker APEXII CCD diffractometer	3082 independent reflections
Radiation source: fine-focus sealed tube	2545 reflections with *I* > 2σ(*I*)
Graphite monochromator	*R*_int_ = 0.032
Detector resolution: 10.0 pixels mm^-1^	θ_max_ = 26.4°, θ_min_ = 2.0°
ω scans	*h* = −7→8
Absorption correction: multi-scan (*SADABS*; Sheldrick, 1996)	*k* = −13→13
*T*_min_ = 0.950, *T*_max_ = 0.967	*l* = −13→13
14052 measured reflections	

### Refinement

**Table:**

Refinement on *F*^2^	Primary atom site location: structure-invariant direct methods
Least-squares matrix: full	Secondary atom site location: difference Fourier map
*R*[*F*^2^ > 2σ(*F*^2^)] = 0.039	Hydrogen site location: inferred from neighbouring sites
*wR*(*F*^2^) = 0.121	H-atom parameters constrained
*S* = 1.06	*w* = 1/[σ^2^(*F*_o_^2^) + (0.0639*P*)^2^ + 0.2592*P*] where *P* = (*F*_o_^2^ + 2*F*_c_^2^)/3
3082 reflections	(Δ/σ)_max_ < 0.001
210 parameters	Δρ_max_ = 0.20 e Å^−^^3^
0 restraints	Δρ_min_ = −0.27 e Å^−^^3^

### Special details

**Table:**

Geometry. All e.s.d.'s (except the e.s.d. in the dihedral angle between two l.s. planes) are estimated using the full covariance matrix. The cell e.s.d.'s are taken into account individually in the estimation of e.s.d.'s in distances, angles and torsion angles; correlations between e.s.d.'s in cell parameters are only used when they are defined by crystal symmetry. An approximate (isotropic) treatment of cell e.s.d.'s is used for estimating e.s.d.'s involving l.s. planes.
Refinement. Refinement of *F*^2^ against ALL reflections. The weighted *R*-factor *wR* and goodness of fit *S* are based on *F*^2^, conventional *R*-factors *R* are based on *F*, with *F* set to zero for negative *F*^2^. The threshold expression of *F*^2^ > σ(*F*^2^) is used only for calculating *R*-factors(gt) *etc*. and is not relevant to the choice of reflections for refinement. *R*-factors based on *F*^2^ are statistically about twice as large as those based on *F*, and *R*- factors based on ALL data will be even larger.

### Fractional atomic coordinates and isotropic or equivalent isotropic displacement parameters (Å^2^)

**Table:**

	*x*	*y*	*z*	*U*_iso_*/*U*_eq_	
C1	0.4597 (3)	0.39262 (18)	0.80434 (17)	0.0376 (4)	
H1A	0.4148	0.3283	0.8637	0.045*	
H1B	0.6026	0.4069	0.8021	0.045*	
C2	0.0821 (3)	0.24463 (18)	0.69445 (17)	0.0380 (4)	
C3	−0.0277 (3)	0.1295 (2)	0.6198 (2)	0.0540 (6)	
H3	0.0283	0.1052	0.5540	0.065*	
C4	−0.2145 (4)	0.0518 (2)	0.6406 (2)	0.0652 (7)	
H4	−0.2850	−0.0234	0.5888	0.078*	
C5	−0.2977 (3)	0.0860 (2)	0.7392 (2)	0.0570 (6)	
H5	−0.4239	0.0335	0.7550	0.068*	
C6	−0.1924 (3)	0.19819 (19)	0.81356 (19)	0.0431 (5)	
H6	−0.2490	0.2197	0.8803	0.052*	
C7	−0.0035 (3)	0.28146 (16)	0.79304 (16)	0.0329 (4)	
C8	0.2416 (2)	0.50716 (17)	0.89652 (15)	0.0301 (4)	
C9	0.4315 (2)	0.51723 (17)	0.84463 (15)	0.0313 (4)	
C10	0.5649 (3)	0.63650 (18)	0.83993 (16)	0.0353 (4)	
H10	0.5326	0.7063	0.8735	0.042*	
C11	0.7565 (3)	0.67060 (18)	0.78813 (16)	0.0355 (4)	
C12	0.7840 (3)	0.6025 (2)	0.68579 (18)	0.0456 (5)	
H12	0.6776	0.5306	0.6474	0.055*	
C13	0.9667 (3)	0.6391 (2)	0.63934 (18)	0.0452 (5)	
H13	0.9814	0.5914	0.5706	0.054*	
C14	1.1265 (3)	0.74520 (18)	0.69391 (17)	0.0364 (4)	
C15	1.1008 (3)	0.81763 (18)	0.79546 (18)	0.0385 (4)	
C16	0.9179 (3)	0.78086 (18)	0.84029 (18)	0.0387 (4)	
H16	0.9014	0.8306	0.9069	0.046*	
C17	1.3474 (3)	0.7111 (2)	0.5578 (2)	0.0515 (5)	
H17A	1.3216	0.6227	0.5804	0.077*	
H17B	1.4859	0.7470	0.5441	0.077*	
H17C	1.2584	0.7119	0.4831	0.077*	
C18	1.2396 (4)	1.0180 (2)	0.9218 (2)	0.0565 (6)	
H18A	1.1360	1.0494	0.8811	0.085*	
H18B	1.3648	1.0894	0.9407	0.085*	
H18C	1.1998	0.9813	0.9973	0.085*	
N1	0.0766 (2)	0.39577 (14)	0.87516 (14)	0.0350 (3)	
H1	−0.0038	0.3951	0.9285	0.042*	
O1	0.23130 (18)	0.59672 (12)	0.96500 (12)	0.0388 (3)	
O2	1.31183 (19)	0.78823 (14)	0.65586 (13)	0.0472 (4)	
O3	1.2663 (2)	0.92142 (15)	0.84312 (17)	0.0639 (5)	
S1	0.31690 (7)	0.33116 (5)	0.65216 (5)	0.04745 (18)	

### Atomic displacement parameters (Å^2^)

**Table:**

	*U*^11^	*U*^22^	*U*^33^	*U*^12^	*U*^13^	*U*^23^
C1	0.0290 (9)	0.0432 (10)	0.0405 (10)	0.0104 (8)	0.0101 (7)	−0.0031 (8)
C2	0.0332 (9)	0.0374 (9)	0.0402 (10)	0.0048 (8)	0.0125 (7)	−0.0046 (8)
C3	0.0504 (12)	0.0454 (11)	0.0578 (13)	0.0012 (9)	0.0200 (10)	−0.0176 (10)
C4	0.0557 (14)	0.0474 (13)	0.0768 (17)	−0.0076 (10)	0.0229 (12)	−0.0235 (11)
C5	0.0394 (11)	0.0432 (11)	0.0765 (16)	−0.0072 (9)	0.0224 (10)	−0.0082 (10)
C6	0.0354 (10)	0.0397 (10)	0.0529 (12)	0.0059 (8)	0.0189 (8)	−0.0014 (8)
C7	0.0286 (9)	0.0314 (9)	0.0375 (9)	0.0070 (7)	0.0084 (7)	−0.0007 (7)
C8	0.0266 (8)	0.0357 (9)	0.0274 (8)	0.0078 (7)	0.0075 (6)	0.0004 (7)
C9	0.0253 (8)	0.0399 (9)	0.0274 (8)	0.0082 (7)	0.0066 (6)	−0.0016 (7)
C10	0.0294 (9)	0.0417 (10)	0.0324 (9)	0.0075 (7)	0.0086 (7)	−0.0038 (7)
C11	0.0293 (9)	0.0406 (10)	0.0351 (9)	0.0072 (7)	0.0102 (7)	0.0014 (7)
C12	0.0316 (10)	0.0527 (12)	0.0405 (10)	−0.0037 (8)	0.0099 (8)	−0.0100 (9)
C13	0.0381 (10)	0.0542 (12)	0.0366 (10)	0.0033 (9)	0.0148 (8)	−0.0115 (8)
C14	0.0284 (9)	0.0411 (10)	0.0388 (10)	0.0065 (7)	0.0142 (7)	0.0015 (8)
C15	0.0304 (9)	0.0337 (9)	0.0481 (11)	0.0034 (7)	0.0141 (8)	−0.0038 (8)
C16	0.0356 (10)	0.0360 (9)	0.0435 (10)	0.0066 (8)	0.0175 (8)	−0.0052 (8)
C17	0.0429 (11)	0.0603 (13)	0.0533 (13)	0.0135 (10)	0.0249 (9)	−0.0065 (10)
C18	0.0549 (13)	0.0365 (10)	0.0703 (15)	0.0005 (9)	0.0220 (11)	−0.0138 (10)
N1	0.0284 (7)	0.0387 (8)	0.0366 (8)	0.0058 (6)	0.0151 (6)	−0.0049 (6)
O1	0.0323 (6)	0.0386 (7)	0.0436 (7)	0.0058 (5)	0.0156 (5)	−0.0079 (5)
O2	0.0335 (7)	0.0492 (8)	0.0555 (9)	0.0030 (6)	0.0238 (6)	−0.0091 (6)
O3	0.0391 (8)	0.0495 (8)	0.0898 (12)	−0.0093 (6)	0.0316 (8)	−0.0320 (8)
S1	0.0377 (3)	0.0537 (3)	0.0418 (3)	−0.0013 (2)	0.0190 (2)	−0.0143 (2)

### Geometric parameters (Å, º)

**Table:**

C1—C9	1.482 (2)	C10—H10	0.9300
C1—S1	1.8058 (19)	C11—C12	1.384 (3)
C1—H1A	0.9700	C11—C16	1.399 (3)
C1—H1B	0.9700	C12—C13	1.386 (3)
C2—C3	1.401 (3)	C12—H12	0.9300
C2—C7	1.402 (2)	C13—C14	1.375 (3)
C2—S1	1.7471 (18)	C13—H13	0.9300
C3—C4	1.367 (3)	C14—O2	1.361 (2)
C3—H3	0.9300	C14—C15	1.398 (3)
C4—C5	1.381 (3)	C15—O3	1.361 (2)
C4—H4	0.9300	C15—C16	1.379 (2)
C5—C6	1.372 (3)	C16—H16	0.9300
C5—H5	0.9300	C17—O2	1.428 (2)
C6—C7	1.397 (2)	C17—H17A	0.9600
C6—H6	0.9300	C17—H17B	0.9600
C7—N1	1.413 (2)	C17—H17C	0.9600
C8—O1	1.229 (2)	C18—O3	1.402 (2)
C8—N1	1.368 (2)	C18—H18A	0.9600
C8—C9	1.490 (2)	C18—H18B	0.9600
C9—C10	1.337 (2)	C18—H18C	0.9600
C10—C11	1.470 (2)	N1—H1	0.8600
			
C9—C1—S1	110.58 (13)	C16—C11—C10	119.12 (16)
C9—C1—H1A	109.5	C11—C12—C13	121.42 (18)
S1—C1—H1A	109.5	C11—C12—H12	119.3
C9—C1—H1B	109.5	C13—C12—H12	119.3
S1—C1—H1B	109.5	C14—C13—C12	120.63 (17)
H1A—C1—H1B	108.1	C14—C13—H13	119.7
C3—C2—C7	118.76 (17)	C12—C13—H13	119.7
C3—C2—S1	115.26 (14)	O2—C14—C13	125.04 (16)
C7—C2—S1	125.97 (14)	O2—C14—C15	115.91 (16)
C4—C3—C2	122.1 (2)	C13—C14—C15	119.05 (16)
C4—C3—H3	118.9	O3—C15—C16	125.11 (17)
C2—C3—H3	118.9	O3—C15—C14	115.09 (15)
C3—C4—C5	119.4 (2)	C16—C15—C14	119.80 (17)
C3—C4—H4	120.3	C15—C16—C11	121.66 (17)
C5—C4—H4	120.3	C15—C16—H16	119.2
C6—C5—C4	119.4 (2)	C11—C16—H16	119.2
C6—C5—H5	120.3	O2—C17—H17A	109.5
C4—C5—H5	120.3	O2—C17—H17B	109.5
C5—C6—C7	122.66 (18)	H17A—C17—H17B	109.5
C5—C6—H6	118.7	O2—C17—H17C	109.5
C7—C6—H6	118.7	H17A—C17—H17C	109.5
C6—C7—C2	117.63 (16)	H17B—C17—H17C	109.5
C6—C7—N1	114.53 (15)	O3—C18—H18A	109.5
C2—C7—N1	127.83 (16)	O3—C18—H18B	109.5
O1—C8—N1	117.55 (14)	H18A—C18—H18B	109.5
O1—C8—C9	120.94 (15)	O3—C18—H18C	109.5
N1—C8—C9	121.42 (15)	H18A—C18—H18C	109.5
C10—C9—C1	124.92 (16)	H18B—C18—H18C	109.5
C10—C9—C8	118.22 (16)	C8—N1—C7	139.93 (14)
C1—C9—C8	116.85 (15)	C8—N1—H1	110.0
C9—C10—C11	127.81 (17)	C7—N1—H1	110.0
C9—C10—H10	116.1	C14—O2—C17	117.20 (15)
C11—C10—H10	116.1	C15—O3—C18	118.52 (15)
C12—C11—C16	117.39 (16)	C2—S1—C1	99.66 (9)
C12—C11—C10	123.42 (17)		
			
C7—C2—C3—C4	−0.5 (4)	C11—C12—C13—C14	−0.2 (3)
S1—C2—C3—C4	−179.5 (2)	C12—C13—C14—O2	179.52 (19)
C2—C3—C4—C5	−1.0 (4)	C12—C13—C14—C15	−1.4 (3)
C3—C4—C5—C6	0.8 (4)	O2—C14—C15—O3	−0.4 (3)
C4—C5—C6—C7	0.8 (4)	C13—C14—C15—O3	−179.59 (19)
C5—C6—C7—C2	−2.2 (3)	O2—C14—C15—C16	−179.92 (17)
C5—C6—C7—N1	176.7 (2)	C13—C14—C15—C16	0.9 (3)
C3—C2—C7—C6	2.0 (3)	O3—C15—C16—C11	−178.25 (19)
S1—C2—C7—C6	−179.12 (15)	C14—C15—C16—C11	1.2 (3)
C3—C2—C7—N1	−176.80 (19)	C12—C11—C16—C15	−2.7 (3)
S1—C2—C7—N1	2.1 (3)	C10—C11—C16—C15	−179.90 (18)
S1—C1—C9—C10	−100.97 (18)	O1—C8—N1—C7	165.2 (2)
S1—C1—C9—C8	80.36 (17)	C9—C8—N1—C7	−18.2 (3)
O1—C8—C9—C10	−21.0 (2)	C6—C7—N1—C8	−177.9 (2)
N1—C8—C9—C10	162.49 (16)	C2—C7—N1—C8	0.9 (4)
O1—C8—C9—C1	157.79 (16)	C13—C14—O2—C17	−5.1 (3)
N1—C8—C9—C1	−18.8 (2)	C15—C14—O2—C17	175.83 (18)
C1—C9—C10—C11	4.2 (3)	C16—C15—O3—C18	−17.0 (3)
C8—C9—C10—C11	−177.11 (16)	C14—C15—O3—C18	163.5 (2)
C9—C10—C11—C12	34.0 (3)	C3—C2—S1—C1	−145.03 (17)
C9—C10—C11—C16	−149.01 (19)	C7—C2—S1—C1	36.02 (19)
C16—C11—C12—C13	2.2 (3)	C9—C1—S1—C2	−85.92 (14)
C10—C11—C12—C13	179.28 (19)		

### Hydrogen-bond geometry (Å, º)

Cg1 is the centroid of the C2–C7 ring.

**Table:**

*D*—H···*A*	*D*—H	H···*A*	*D*···*A*	*D*—H···*A*
C12—H12···S1	0.93	2.76	3.605 (2)	151
N1—H1···O1^i^	0.86	2.12	2.967 (2)	170
C6—H6···O1^i^	0.93	2.52	3.318 (2)	144
C5—H5···O3^ii^	0.93	2.46	3.377 (3)	167
C17—H17*C*···*Cg*1^iii^	0.96	2.84	3.724 (2)	154
C18—H18*A*···*Cg*1^iv^	0.96	2.90	3.841 (3)	166

Symmetry codes: (i) −*x*, −*y*+1, −*z*+2; (ii) *x*−2, *y*−1, *z*; (iii) −*x*+1, −*y*+1, −*z*+1; (iv) *x*+1, *y*+1, *z*.
